# Assessing Patient Trust in Automation in Health Care Systems: Within-Subjects Experimental Study

**DOI:** 10.2196/48584

**Published:** 2024-08-06

**Authors:** Matthew Nare, Katherina Jurewicz

**Affiliations:** 1 School of Industrial Engineering and Management Oklahoma State University Stillwater, OK United States

**Keywords:** automation, emergency department, trust, health care, artificial intelligence, emergency, perceptions, attitude, opinions, belief, automated, trust ratings

## Abstract

**Background:**

Health care technology has the ability to change patient outcomes for the betterment when designed appropriately. Automation is becoming smarter and is increasingly being integrated into health care work systems.

**Objective:**

This study focuses on investigating trust between patients and an automated cardiac risk assessment tool (CRAT) in a simulated emergency department setting.

**Methods:**

A within-subjects experimental study was performed to investigate differences in automation modes for the CRAT: (1) no automation, (2) automation only, and (3) semiautomation. Participants were asked to enter their simulated symptoms for each scenario into the CRAT as instructed by the experimenter, and they would automatically be classified as high, medium, or low risk depending on the symptoms entered. Participants were asked to provide their trust ratings for each combination of risk classification and automation mode on a scale of 1 to 10 (1=absolutely no trust and 10=complete trust).

**Results:**

Results from this study indicate that the participants significantly trusted the semiautomation condition more compared to the automation-only condition (*P*=.002), and they trusted the no automation condition significantly more than the automation-only condition (*P*=.03). Additionally, participants significantly trusted the CRAT more in the high-severity scenario compared to the medium-severity scenario (*P*=.004).

**Conclusions:**

The findings from this study emphasize the importance of the human component of automation when designing automated technology in health care systems. Automation and artificially intelligent systems are becoming more prevalent in health care systems, and this work emphasizes the need to consider the human element when designing automation into care delivery.

## Introduction

Delays in care can come from any number of factors that influence the health care work system, including technology and other automated computing tools that support patient care [1]. Understanding the dynamics between humans and technology, such as automated technology, allows designers to develop the appropriate equipment for completing patient care tasks in different clinical environments. Advancements in technology (eg, artificial intelligence and smart medical devices) bring new applications in health care with the intent to improve the delivery of care [2]. New technologies that are currently being implemented in health care systems change the way hospitals maintain a patient’s health record [3] and surgeons conduct surgery [4,5] and assist with patient mobility [6]. Advancements in smart health care technology have allowed health care work systems to become increasingly interconnected. The adoption of electronic health records or electronic medical records opened communication pathways between patients and clinicians [7]. Videoconferencing capabilities have advanced the opportunities for remotely conducting appointments and inpatient communication [8]. With the increased use of technology within health care work systems, it is imperative to understand the short- and long-term effects of that technology.

One area where technology has made significant strides is with automation. Automation has been defined in many ways, but the consistent theme across the definitions is a technology completing a task for humans [9,10]. Parasuraman and Riley [10] stated as part of their definition of automation that “what is considered automation will therefore change with time.” The evolution of automation is portrayed through the types of technologies and industries integrating automation into daily workflows. However, in order for automated technology to be effectively used, it is important for the human user to trust the output of the automated system. Trust, like automation, has received many operational definitions depending on the context and field [11,12]. Development of trust in automation is a function of many factors including personality and system performance. However, humans are known to overtrust or become overreliant on automation, which has been known to cause unwanted outcomes [9]. Automation must be designed to promote trust in a way that adapts to its users’ needs while lending itself to allow for the appropriate amount of reliance [9,13]. Chiou and Lee [13] described the importance of designing adaptive and resilient automation to improve the trust dynamics between automation and its users. Automation errors occur when there are discrepancies between the amount of trust and the capabilities of the system [9].

There are several areas within the health care system that can benefit from automated technologies including high-risk environments, such as emergency medicine. Emergency medicine is a dynamic work environment, and there is a constant turnover of patients presenting with varying symptoms ranging in severity in emergency medicine. Health care providers use a triage system to prioritize arriving patients based on the severity of the diagnosis. Health conditions such as myocardial infarctions (ie, heart attacks) and cerebrovascular attacks (ie, strokes) require immediate medical intervention. A delay in care can have irrevocable effects on a patient’s health. Heart disease is a leading cause of death in the United States, and it is estimated over 800,000 people in the United States experience a heart attack every year [14]. Despite the prevalence of myocardial infarctions in the United States, the symptoms continue to be missed within emergency departments (EDs) [15]. Integrating automation into EDs capable of making an accurate assessment of a patient’s symptoms has the potential to assist one aspect of a clinician’s patient intake workflow.

Health care automation has the capability of improving clinical outcomes and hospital environments; however, for this to occur, automation must be designed in a way that allows for seamless integration into the work environment. To improve both health care providers’ and patients’ acceptance of automated technology, automation must be designed to promote the appropriate level of trust within its users [9,13]. The first step to designing automation that promotes the appropriate level of trust is to understand which aspects of the technology enhance or detract trust in all health care providers responsible for operating or interpreting the results of automated technology (ie, physicians and nurses). While the health care providers are the primary user group when it comes to health care automation, it is vital to understand the patient’s perspective, as patients may have reservations for integrating automation into health care work systems [16]. Although health care consists of both providers and patients, there is little literature investigating the automation needs for both parties. Therefore, the objective of this study was to study the effect of automation mode on a patient’s trust in a cardiac risk assessment tool (CRAT). A secondary objective was to study the effect of the severity of symptoms on a patient’s trust in the CRAT.

## Methods

### Study Design

A within-subjects experimental study was performed to assess a patient’s trust in the CRAT. The CRAT was designed to have three automation modes: (1) no automation, (2) automation only, and (3) semiautomation. No automation was defined as a physician acting as the cardiac risk assessor without any technology, the automation-only condition was defined as a fully automated risk assessment tool that had no human intervention, and the semiautomation condition consisted of the risk assessment tool that was validated by the experimenter.

### Participants

In total, 12 participants were recruited from undergraduate and graduate students at Oklahoma State University and the general Stillwater, Oklahoma community to participate in this study.

### Ethical Considerations

This study received exempt status from the Oklahoma State University Institutional Review Board (IRB-22-391-STW). All participants completed the informed consent process with the experimenter, and all data were anonymized. Each participant received a $10 Amazon gift card for their time.

### Equipment and Materials

**Figure 1:**
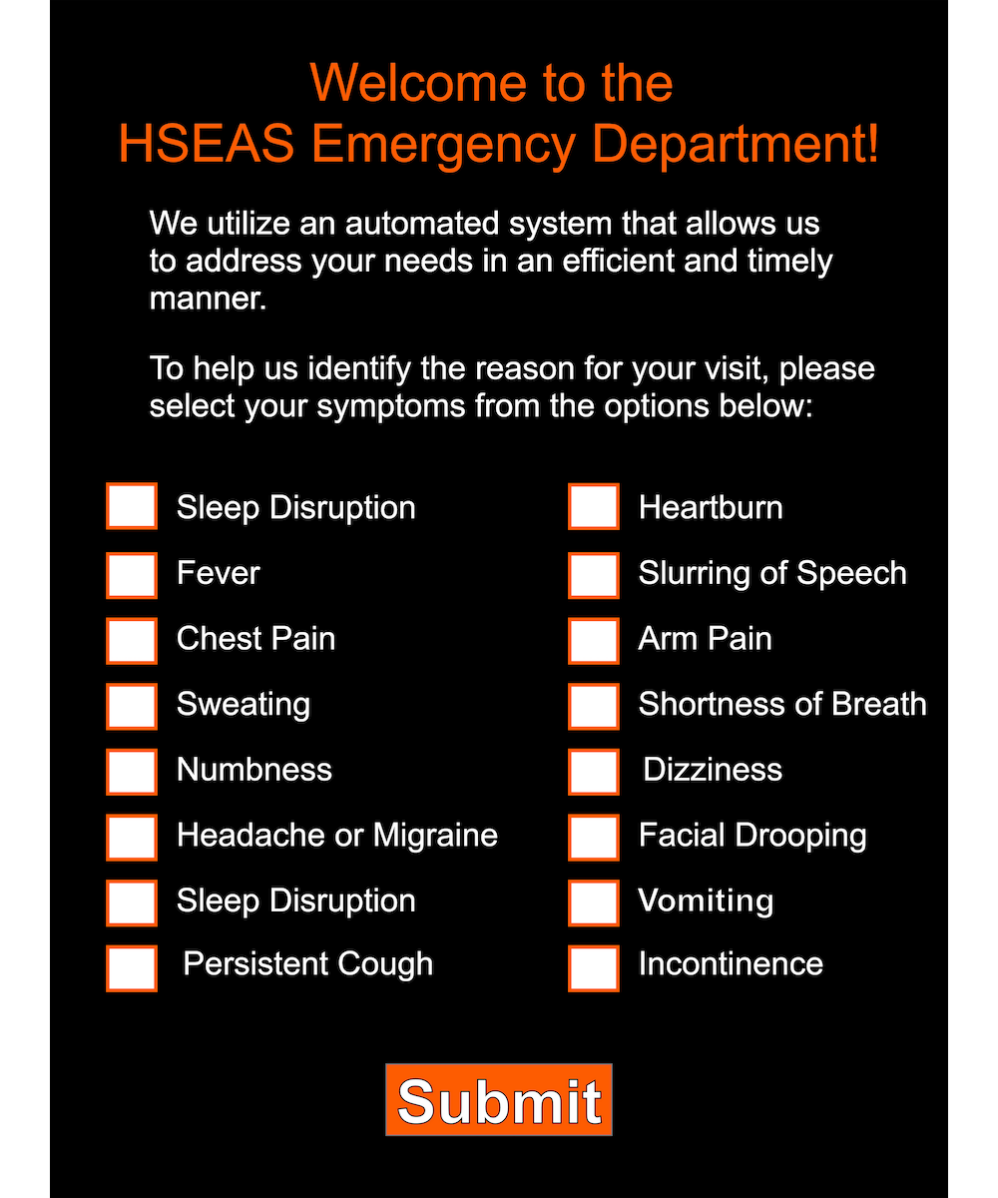
Digital symptom application prototype showing 16 symptom options and checkboxes.

**Figure 2:**
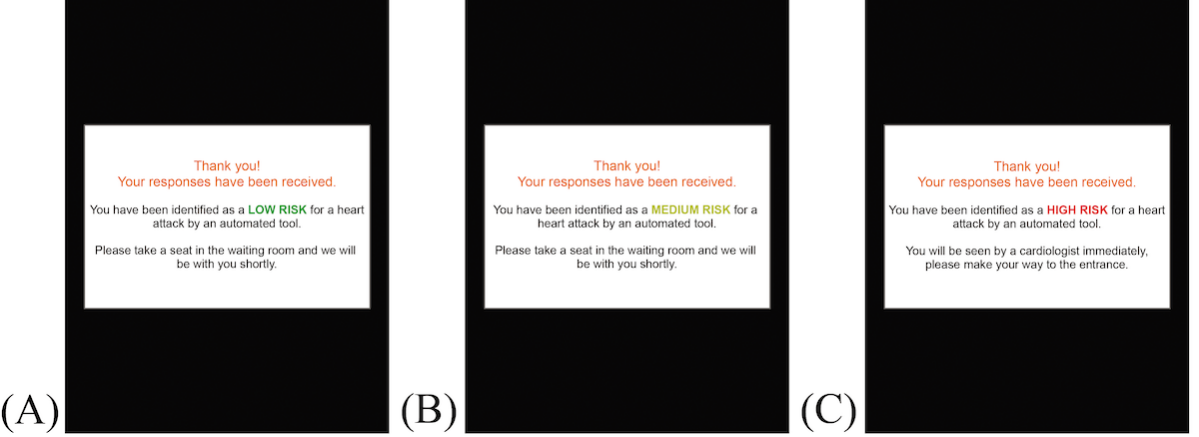
Digital symptom application prototype risk classification message for each risk classification level: (A) low, (B) medium, and (C) high risks.

A digital application prototype was developed with Adobe XD (Adobe). The prototype displayed 16 potential health status symptoms with a touch-activated check box (Figure 1). Participants received a risk classification after checking their symptoms and selecting the submit button (Figure 2). Participants navigated the application using a Microsoft Surface tablet (Microsoft Corp).

### Procedure

Upon arriving, the participants were welcomed to the Human-Systems Engineering Applied Statistics (HSEAS) Lab, and the informed consent process was completed with the participants. Participants were then seated at a table facing the computer monitor to complete the demographics questionnaire to collect data about the participants’ personal characteristics (eg, school status, age, and ethnicity), personal or family professional health care experience, experience as a patient or family member in an ED, the tendency to research medical information on the web, and subjective ratings on their quality of health.

Participants completed a training session on heart attack symptoms by reading a document titled “What are the warning signs of a heart attack?” [17]. This document ensured that participants understood the symptoms of a heart attack before completing the experiment. Participants were then relocated to a simulated waiting room area. For the remainder of the experimental session, the participant assumed the role of a patient arriving at the HSEAS Lab ED (ie, the simulated waiting room at the HSEAS Lab), and the experimenter performed the role of the health care provider. Each participant gave trust ratings for 3 different scenarios for each mode of information.

Automation mode (eg, automation only, no automation, or semiautomation) was presented across 3 blocks. In the automation-only condition, the participant used the tablet to independently submit their symptoms and only received their risk classification from the tablet. The semiautomation condition consisted of the patient (ie, the participant) working with the health care provider (ie, the researcher) to submit the correct symptoms and then receiving a risk classification from the tablet that was confirmed by the health care provider. Thus, the experimenter and the participant worked together to enter the symptoms. In the no automation condition, participants verbally stated their symptoms to the health care provider and were verbally provided their risk classification. The automation modes (eg, automation only, no automation, or semiautomation) were based on the trust in the automation framework presented by Chiou and Lee [13].

The order of the blocks, and thus automation mode, was counterbalanced across the participants. Participants were then classified as high risk, medium risk, or low risk based on the symptoms provided in the symptom severity scenarios (Table 1). Participants were provided the written scenario (ie, symptom severity scenario) that included the present symptoms and a subjective description of how they were feeling as a result of those symptoms. The risk levels were randomized within the block; therefore, each participant completed 9 different scenarios for each automation mode and risk level classification.

**Table 1:** Risk classification symptoms and subjective description.

Risk severity	Present symptoms	Lacking symptoms	Subjective description
High risk	Sweating Shortness of breath Severe chest pain Nausea	None	Feel “like an elephant is sitting on my chest”
Medium risk	Sweating Nausea Mild chest discomfort	Severe chest pain Shortness of breath	Feel “like there is a mild pain in my chest”
Low risk	Rapid heart rate	Shortness of breath Sweating Severe chest pain Nausea	Feel “like something is wrong because my heart is beating fast”

During this study, the prototype provided the correct risk classification for each symptom presentation scenario using a “Wizard of Oz” technique; thus, the automation was always correct in the risk classification. Participants were informed that there may be errors in the risk classification and that the submitted symptoms will help fine-tune the output of the CRAT. At the end of each scenario, participants were asked to provide their rating of trust in the provided risk classification on a scale of 1 to 10 (1=absolutely no trust and 10=complete trust); therefore, each participant had 1 trust rating for every combination of automation mode and risk level for a total of 9 ratings per participant. The participants’ subjective ratings of trust were collected following a similar approach presented in previous literature on measuring trust in automation via Likert scale ratings [18-20].

### Data Analysis

A mixed effects linear regression model, with trust as the dependent variable, was fitted to the data, and stepwise deletion was performed until the best fit linear regression model was determined. Tukey contrasts were calculated to perform pairwise comparisons for all variables with multiple levels in the regression model. All statistical analyses were performed in RStudio (version 2024.04.1+748; Posit Software, PBC) using the *lme4* package (version 1.1-26) [21] and the *multcomp* package (version 1.4-16) [22].

## Results

A majority of participants identified as female (n=8), and the rest identified as male (n=4). The age of participants ranged between 18 and 52 (mean 24.56, SD 9.10) years. In total, 6 participants were current students pursuing either an undergraduate or graduate degree at Oklahoma State University, and 6 participants were members of the greater Stillwater community (ie, nonstudents). There was 1 participant who reported working in the medical field, and 7 of the participants reported having family members who worked in the medical field. A majority of 11 participants reported having been to a doctor in the past year with 10 participants having between 2 and 4 doctor visits in the past year. In total, 7 participants had previously visited an ED as a patient and 6 participants had visited an ED with a family member. Overall, 3 participants had visited an ED in the past year. The frequency for all trust ratings and the percentage of the total response are provided in Table 2. The average trust rating overall across all conditions was 8.57 (SD 1.56).

**Table 2:** Summary of trust ratings by the frequency of occurrence (n=108).

Trust rating	Values, n (%)
0	—^a^
1	—
2	—
3	1 (0.9)
4	1 (0.9)
5	3 (2.8)
6	6 (5.6)
7	16 (14.8)
8	13 (12)
9	28 (25.9)
10	40 (37)

^a^Not available.

The average trust ratings for automation modes and risk levels are provided in Table 3. Between automation modes, the lowest trust ratings were provided for automation-only condition (mean 7.68, SD 1.42) and the highest trust ratings for the semiautomated condition (mean 9.08, SD 1.44). Overall, the highest trust ratings were for the high risk level in the semiautomated condition (mean 9.58, SD 0.86), and the lowest trust ratings were for the medium risk in the automation-only condition (mean 6.91, SD 1.38).

**Table 3:** Summary of trust rating.

Automation mode and risk level			Average trust rating, mean (SD)		Overall average trust rating, mean (SD)
**Automation only**					
	Low	8.08 (1.19)		7.86 (1.42)	
	Medium	6.91 (1.38)		7.86 (1.42)	
	High	8.58 (1.11)		7.86 (1.42)	
**Semiautomation**					
	Low	9.25 (1.01)		9.08 (1.44)	
	Medium	8.42 (1.93)		9.08 (1.44)	
	High	9.58 (0.86)		9.08 (1.44)	
**No automation**					
	Low	8.67 (1.93)		8.78 (1.53)	
	Medium	8.50 (1.38)		8.78 (1.53)	
	High	9.17 (1.07)		8.78 (1.53)	

The mixed effects model showed that the random effect was nonsignificant in explaining any additional variability; however, to provide the most accurate representation of the data, the random effect was included in the final model. The results of the final regression model are shown in Table 4. The Akaike information criterion for the linear mixed effects model was 391.155. There was no interaction term included in this model.

**Table 4:** Linear regression table for the final linear mixed effects model.

Coefficients	Estimate (SE)	*t* test (*df*)	*P* value
Intercept	8.791 (0.372)	23.636 (92)	<.001
Automation only	–1.222 (0.316)	–3.869 (92)	<.001
No automation	–0.306 (0.316)	–0.967 (92)	.34
High risk level	1.167 (0.316)	–3.693 (92)	<.001
Low risk level	0.722 (0.316)	2.286 (92)	.02
EDPatientYes^a^	–0.578 (0.351)	–1.647 (10)	.13

^a^ED: emergency department.

Automation mode (ie, automation only; *P*<.001) and risk level (ie, low and high risk; *P*=.02 and *P*<.001) were significantly associated with the participants’ trust ratings. The Tukey pairwise comparisons for automation mode indicated that there were significant differences in the participants trust between no automation and automation only (*P*=.03) as well as automation only and semiautomation (*P*=.002).

Trust in the risk classification was also significantly lower when participants were classified as medium risk compared to a high-risk classification (*P*=.004). The Tukey pairwise comparisons showed no other significant differences between risk classification levels.

## Discussion

### Principal Findings

As automation becomes more integrated into health care work systems, it is important to understand the implications of automation on the patients and clinicians. Investigating patients’ perceptions toward the use of automated technology, such as their trust, represents one such area of research. The purpose of this study was to investigate patients’ perceptions of an automated CRAT prototype developed to improve the triage process for patients with cardiac symptoms arriving at an ED. Findings from this study indicate that the factors significantly associated with trust were the automation mode and risk level.

Participants reported significantly higher ratings of trust in the risk classification when a human participated (ie, no automation and semiautomation conditions) in passing on the information to the patient compared to when the risk classification was only presented with the CRAT (ie, automation-only condition). These findings support the value of keeping the human, both patients and clinicians, involved when integrating health care automation into hospitals. Health care traditionally includes a close patient-provider interaction [2]; however, as technology has evolved, patient-provider interactions adapt to incorporate more technology into patient care. Unlike other industries where the roles of humans decrease with automation, health care technology requires more human involvement to properly monitor large amounts of data introduced during Health Care 4.0 [2].

There was a significant difference in trust ratings for the high-risk scenarios and the medium-risk scenarios. The high-risk scenario provided symptoms that were easily interpreted by the participants; however, the symptoms for the medium-risk scenario were more ambiguous. While the high-risk scenario incorporated all the traditional heart attack symptoms (eg, complaints of severe chest pain, shortness of breath, and nausea), the medium-risk scenario presented symptoms with multiple interpretations (eg, mild chest discomfort could be interpreted as heartburn). The difference in trust ratings between the high- and medium-risk scenarios suggests that there may be a relationship between the quality of information used by the automation to draw its conclusion and trust. Given a participant’s lack of trust based on their individual differences, it is important to understand how ambiguous situations, such as the medium-risk scenario, can challenge their trust in the output [23,24]. It is possible that the participants trusted the automation more during the clear scenarios compared to the ambiguous scenario because of their own automation complacency or bias [25]. The medium-risk scenario may have been ambiguous since the participant could have interpreted the symptoms as more or less severe; therefore, it may have required more thought on whether the risk classification was correct. The ambiguity may have increased the level of doubt the participants experienced in the CRAT’s output as trust is dynamic and constantly changing as different situations unfold [11,13].

All of the components of the health care work system are interconnected; therefore, this means that the performance of one work system element influences the other elements [1]. If we remove the human operator from the work system, there may be a negative effect on the care processes, which influence patient and organization outcomes. With hospitals focused on patient-centered care and patient outcomes, it is important to consider how patients perceive the technology used in care environments [8,26]. As new technology, such as artificial intelligence and automated technology, is integrated into health care work systems, it is important to appropriately design the interactions between humans and technology, especially designing for trust in automation [13].

The use of automated technology to assist in the intake and triage of patients has the potential to simultaneously support providers’ work and the patient experience. The full extent of this potential is dependent on developing automation capable of providing trustworthy and accurate information to both parties. Chiou and Lee [13] described a framework where automation and the human operator were influenced by the context of repeated interactions between single human operator and one form of automation. In health care, interaction with automation may include simultaneous use effects for both patients and clinicians. Patients are primarily on the receiving end for treatments provided by automation, while clinicians are responsible for the actions that lead to automation-driven treatment outcomes. Given the clinician and patient are both involved in the treatment process, both parties experience consequences when errors occur. During one ED experience, patients may receive treatment from several devices from several clinicians, and clinicians may operate several devices while delivering care to several different patients. Automation needs to be designed in a way to mitigate the risk of errors occurring due to the work pressures of clinical environments.

### Limitations

This study emphasized the importance of humans in the development of automated technology for health care, and there are several areas of future work. The CRAT, while developed based on input from health care providers and material, was not validated as being a system that would be integrated into an ED, which means there is an opportunity to continue to develop the CRAT’s viability and usability using ED provider feedback. Future work should also investigate the trust and perceptions of ED providers to understand how the use of automated technology such as the CRAT changes provider workload and care quality from their perspective as well as further investigations into patients’ trust. This study focused on studying automation mode; however, individual differences, such as language, ethnicity, race, or cultural diversity, all of which may influence how a person trusts technology, may be important to study in future work. This study has a limited sample size of 12 participants, and we would like to continue this work with more participants in the future. Additionally, improving the diversity of future studies, such as expanding to a wider age range and number of participants, will provide the opportunity to gain more insights into the challenges of trusting automation.

To our knowledge, this is the first study investigating trust in a risk assessment tool such as the one studied here. The small sample size and simulated setting (ie, a simulated ED rather than a live ED) were selected to test the viability of this work while being able to remove the risk of negative impact on patient care in a live ED. Selecting heart attack risk as the diagnosis of focus for this study allowed for trust ratings to be gathered on an adverse health event, which has widely known consequences across the general populace. With that in mind, future work should investigate the trust in risk assessment tools for other adverse diagnoses (eg, neurological events). Furthermore, as automation is integrated into health care systems, designing for failures in automation (eg, misclassification of heart attack risk) should be studied in future work as well as the effect of automation failures on trust. Future investigation into the effect of automation failures is vital to understand how trust changes within a health care environment, where each care decision carries positive and negative outcomes for the patient and the provider. This work offers a foundation for trust in automation within health care to build on as future research with larger, more diverse sample sizes and greater ecologically valid environments to improve the generalizability of this work.

### Conclusions

Automation and artificial intelligence are used to support clinical decision-making and ease the workload of health care providers. Using automation and artificial intelligence to support accurate and time-efficient decisions demonstrates new opportunities for advancing patient-centered care. Designing trustworthy automation can provide additional support when a clinical environment is understaffed or without access to specialty care such as rural clinics. This work represents an important first step at quantifying how patients trust automated technology within health care. The results from this study indicate that patients trust technology most when there is a combination of human and automation interaction throughout the care process and when there is little to no ambiguity based on their symptoms. Keeping the human involved in the process enhances the transparency of the automation with the patient, which can improve trust. Designing automation with the patient and clinician in mind is important when attempting to integrate automation into health care work environments, where errors can lead to irrevocable consequences such as patient death.

## Abbreviations

CRAT: cardiac risk assessment tool
ED: emergency department
HSEAS: Human-Systems Engineering Applied Statistics
